# Establishment of multifactor predictive models for the occurrence and progression of cervical intraepithelial neoplasia

**DOI:** 10.1186/s12885-020-07265-7

**Published:** 2020-09-29

**Authors:** Mengjie Chen, He Wang, Yuejuan Liang, Mingmiao Hu, Li Li

**Affiliations:** Guangxi Medical University affiliated Cancer Hospital, NO.71 Hedi Road Qingxiu Square, Nanning City, Guangxi Province China

**Keywords:** Cervical intraepithelial neoplasia, Cervical cancer, Random forest model, Bioinformatics

## Abstract

**Background:**

To study the risk factors involved in the occurrence and progression of cervical intraepithelial neoplasia (CIN) and to establish predictive models.

**Methods:**

Genemania was used to build a gene network. Then, the core gene-related pathways associated with the occurrence and progression of CIN were screened in the Kyoto Encyclopedia of Genes and Genomes (KEGG) database. Real-time fluorescence quantitative polymerase chain reaction (RT-qPCR) experiments were performed to verify the differential expression of the identified genes in different tissues. R language was used for predictive model establishment.

**Results:**

A total of 10 genes were investigated in this study. A total of 30 cases of cervical squamous cell cancer (SCC), 52 cases of CIN and 38 cases of normal cervix were enrolled. Compared to CIN cases, the age of patients in the SCC group was older, the number of parities was greater, and the percentage of patients diagnosed with CINII+ by TCT was higher. The expression of TGFBR2, CSKN1A1, PRKCI and CTBP2 was significantly higher in the SCC groups. Compared to patients with normal cervix tissue, the percentage of patients who were HPV positive and were diagnosed with CINII+ by TCT was significantly higher. FOXO1 expression was significantly higher in CIN tissue, but TGFBR2 and CTBP2 expression was significantly lower in CIN tissue. The significantly different genes and clinical factors were included in the models.

**Conclusions:**

Combination of clinical and significant genes to establish the random forest models can provide references to predict the occurrence and progression of CIN.

## Background

Cervical cancer is a female malignant tumor, and it has the second highest morbidity rate and the third highest mortality rate in the world [1]. Cervical intraepithelial neoplasia (CIN) is a precancerous lesion that precedes invasive cervical cancer. Persistent high-risk human papillomavirus (HPV) infection is one of the main causes of cervical cancer and CIN, but individual genes and other clinical factors also have an important impact on the progression of CIN [2]. Cervical cytology, HPV testing, colposcopy and cervical biopsy histopathology are widely used clinically to screen for CIN. The occurrence and outcome of CIN are closely related to genes, vaginal microecology, environment and other factors. CIN is classified into CINI, CINII, and CINIII grades. Sixty percent of CINI grades can regress spontaneously, and only 10 and 1% of them progress to CINIII and cervical invasive carcinoma, respectively. CINII grade has a 5% possibility of developing cervical invasive cancer, but the probability of CINIII progressing to cervical invasive cancer is higher than 12% [3].

The occurrence and progression of CIN is a very complex and multifactorial process. Cervical cytology analyses, HPV tests, colposcopies and cervical biopsy histopathology analyses are widely used in the clinic to screen for cervical intraepithelial neoplasia (CIN). However, the cytological diagnosis, HPV-DNA detection, pathology and single gene analysis are not capable of predicting the outcome of CIN. A large number of women every year may receive unnecessary treatment or may delay treatment. Therefore, combining the clinical features and significantly differentially expressed genes in CIN patients, a multifactor predictive model can be produced to more accurately predict the occurrence and progression of CIN, enabling the shunting of CIN patients according to risk factors for progression and the development of individualized treatment plans for different patients.

## Methods

### Selection of genes

The progression of CIN is related to signaling pathways such as the Wnt signaling pathway, the endocytosis signaling pathway and the *Vibrio cholerae* infection pathway. Among the genes of these pathways, CCND2, CDKN2A, CADM1, CCL2, CTNNB1, ERBB2, PHGDH, TP53BP1, TP63, TGFBR2, EGFR, PRKCB, SH3KBP1, KDELR1, NFATC1, PPP2R5D, HSPA6, PIKFYVE, RABEP1, TJP2, PIK3CA, PRKCI, PTGS2, STK11, FOXO1, TP53, MYC, IMP3 and MAPK1 are known to interact, and these genes may also be related to the occurrence and progression of CIN [4]. Genemania was used to construct a gene network and explore the relationships among CCND2, CDKN2A, CADM1, CCL2, CTNNB1, ERBB2, PHGDH, TP53BP1, TP63, TGFBR2, EGFR, PRKCB, SH3KBP1, KDELR1, NFATC1, PPP2R5D, HSPA6, PIKFYVE, RABEP1, PRKCI, PTGS2, STK11, FOXO1, TP53, PIK3CA, MYC, IMP3 and MAPK1. The genes at the core of the network were selected, and the signaling pathways associated with these genes were further explored in the KEGG database to find other genes in the same pathway. The genes located in the same signaling pathway and jointing multiple signaling pathways were selected for study.

### Gene assays

#### Reagents and materials

Sample protector for DNA/RNA (Takara 9750), RNA iso Plus (Takara 9109), PrimeScript™ RT reagent Kit with gDNA Eraser (Takara RR047A),SYBR®Premix Ex Taq™II (Takara RR820A) and primers were obtained from the Takara (Japan). A QuanStudio5 thermal cycler was purchased from Thermo Fisher (America). All experiments were performed according to the manufacturer’s instructions.

#### Clinical data and specimens

A total of 120 cases were used, and specimens were obtained from the Gynecology Oncology Department of Guangxi Medical University Affiliated Cancer Hospital. The clinical features included age, gravity, parity, and HPV status. Normal cervical tissues were taken from patients who underwent a hysterectomy for uterine leiomyoma. CIN tissues were taken from postoperative cervical specimens obtained after cervical cold knife conization, and SCC tissues were collected from the tumor specimens of radical hysterectomies. Specimens were put in a sample protector solution immediately and then were frozen and stored at − 80 °C as soon as possible. A total of 38 normal cervical tissues, 52 CIN tissues and 30 cervical squamous carcinoma tissues were collected. The status of all specimens included in the study was confirmed by pathology diagnosis.

#### RT-qPCR

Total RNA was extracted from tissues by TRIzol. The RNA concentration was 800–1500 ng/μl, and the optical density value (OD) was 1.7–2.0. One hundred nanograms of total RNA was reverse transcribed to generate cDNA. RT-qPCR was performed using a SYBR Green dye method. RT-qPCR reaction conditions were as follows: 95 °C 30 s for 1 cycle, 95 °C 5 s, 60 °C 30 s for 10 cycles, 95 °C 30 s for 1 cycle, and 95 °C 5 s, 60 ° 30 s for 40 cycles. The experiments were repeated 3 times. An absolute quantitative method was used for the experiments. The following formula was used to calculate expression: copies of target genes/copies of reference genes. β-actin served as the reference gene. The sequences of primers were showed in Table 1.

#### Statistical analyses

SPSS 22.0 software was used to perform statistical analysis. The data are expressed as the mean ± standard deviation, and the group rank sum test was used for comparisons between groups. Chi-square tests were used for comparisons between classification data groups. Multivariate analysis uses binary logistic regression. A *P*-value less than 0.05 was considered to be significantly different.

#### Random Forest models

The randomForest package of Rstudio software was used to establish random forest models. The random numbers were generated by the seed.set function. Of the cases, 50% (60 cases) were randomly selected as the training set, and 50% (60 cases) were used as the test set. Using importance to evaluate the weight of each variable in the model, the mean decreased accuracy indicated a decrease in accuracy after variable substitution, and the mean decreased Gini indicated a decrease in the Gini coefficient after variable substitution. The larger the value was, the more important the variable was. The overall error of the model was evaluated by out-of-bag error (OOB error). The diagnostic effect of the model was evaluated by AUC and accuracy.

## Results

### Candidate genes

**Fig. 1:**
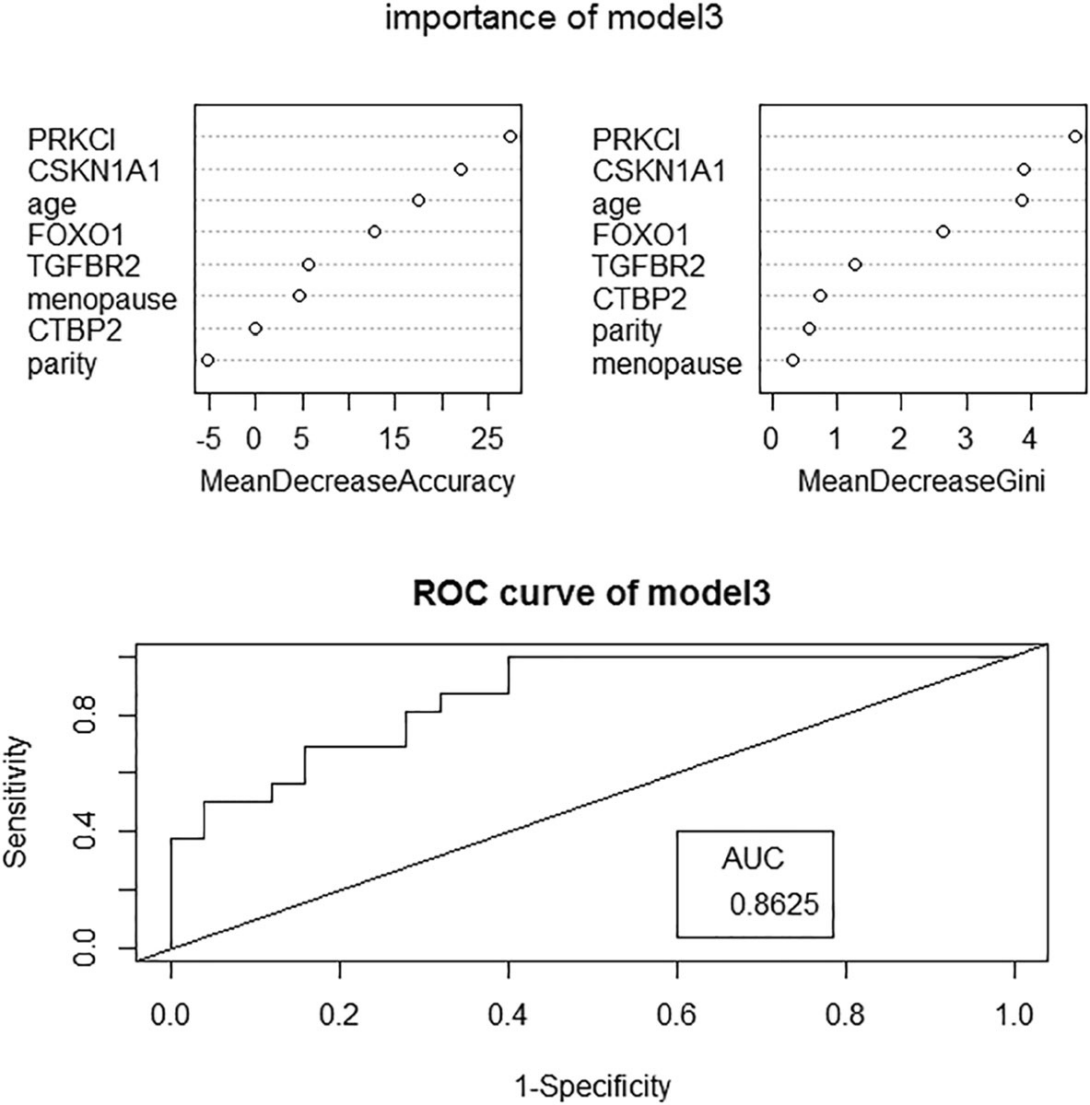
the weight of factors in model3. the ROC curve of model3

Genemania was used to build a gene network and explore the interactions between genes (Fig. 1). In the network, CCND2, CTNNB1, PRKCI, PIK3CA, FOXO1, MUC2, TGFBR2, TP73, CSNK1A1, CTBP2, AK5, GRHPR, KDELR3, and NCOA2 were located at the core of the network. Among them, AK5, GRHPR, KDELR3 and NCOA2 have not been reported to be related to human solid tumors. Considering that the genes worked through signaling pathways, the pathways containing the highest number of genes were selected for study. Most genes in two pathways were found in the HPV infection signaling pathway and Hippo signaling pathway. The genes in the HPV infection pathway were CCND2, CTNNB1, PRKCI, FOXO1 and CTBP2. The genes in the Hippo signaling pathway were CCND2, CTNNB1, PRKCI, TGFBR2 and TP73. The genes coexisting in both pathways were CCND2, CTNNB1 and PRKCI. Additionally, previous reports suggest that MUC2 and TGFBR2 may be related to the progression of CIN [4], so MUC2 was included in this study. CSNK1A1 and CTBP2 are at the core of the gene network, and it has been shown that these genes are correlated with the occurrence and development of various solid tumors. Ten candidate genes were finally identified in this study: TGFBR2, CSKN1A1, PRKCI, FOXO1, CTNNB1, CCND2, MUC2, TP73, CTBP2, and PIK3CA. This study will explore the role of these genes in the occurrence and progression of CIN.

### Clinical features and gene expression

A total of 120 cases were used in this study: 38 normal cervix cases, 52 cases of CIN and 30 cases of SCC. The clinical characteristics of the cases were as follows.

Compared to the CIN group, the patients in the SCC groups were older (*P* = 0.000) and had more parity (*P* = 0.017), and the percentage of premenopausal cases (*P* = 0.000) were significantly higher. The expression levels of TGFBR2, FOXO1, CSKN1A1, PRKCI, and CTBP2 in CIN and SCC tissue samples were significantly different. The expression levels of TGFBR2, CSKN1A1, PRKCI, and CTBP2 were significantly upregulated in the SCC group, while FOXO1 was expressed at significantly lower levels in the SCC group (Table 1). The clinical factors related to the progression of CIN were older age, more parity, and premenopause; the significantly upregulated genes in this group were TGFBR2, FOXO1, CSKN1A1, PRKCI, and CTBP2.

Compared to the normal group, the proportion of CIN cases with HPV infection (*P* = 0.000) and TCT-diagnosed CINII+ (P = 0.000) was significantly higher than that of the normal group. FOXO1 expression levels were significantly higher in the CIN group, while TGFBR2 and CTBP2 were significantly lower in the CIN group (Table 2). The clinical factors related to the occurrence of CIN were HPV infection and TCT-diagnosed CINII+, and the significantly upregulated genes were TGFBR2, FOXO1 and CTBP2.

**Table 1:** The clinical features and genes expressed in the CIN and SCC groups

	CIN	SCC	Z/X^2^	P
**age**	41.71 ± 9.31	49.87 ± 10.03	−3.575	**0.000**
gravidity	3.46 ± 1.84	4.00 ± 1.44	−1.720	0.085
**parity**	1.67 ± 1.34	2.30 ± 1.34	−2.377	**0.017**
HPV infection				
positive	47	28	/	1.000
negative	5	2		
**menopause**			13.610	**0.000**
yes	9	17		
no	43	13		
TCT				0.109
CINII-(Normal/ASCUS)	16	4	/	
CINII+(LSIL/HSIL/SCC)	36	26		
CTNNB1	0.88 ± 0.24	4.23 ± 2.47	−1.637	0.102
**TGFBR2**	665.56 ± 357.49	15,156.14 ± 9065.91	−3.023	**0.003**
**FOXO1**	0.50 ± 0.30	0.10 ± 0.05	−3.110	**0.002**
**PRKCI**	0.56 ± 0.11	5.49 ± 2.66	−4.130	**0.000**
**CSKN1A1**	135.69 ± 81.39	4062.52 ± 1658.02	−3.783	**0.000**
TP73	75.39 ± 21.31	408.60 ± 145.24	−1.714	0.087
MUC2	1.89 ± 1.26	1.35 ± 0.70	−1.271	0.204
PIK3CA	7.28 ± 1.98	8.60 ± 3.22	−0.053	0.958
CCND2	1.40 ± 0.72	0.53 ± 0.25	−1.078	0.281
**CTPB2**	16.59 ± 6.14	599.16 ± 375.43	−2.089	**0.037**

### Logistic analysis of risk factors for CIN progression and occurrence

To explore the risk factors for the occurrence and progression of CIN, a logistic regression analysis was conducted. The factors related to the progression of CIN that were identified in part 3.2, including older age, premenopause and multiple parity as well as significantly differentially expressed genes TGFBR2, FOXO1, CSKN1A1, PRKCI, and CTBP2, were included in the univariate logistic analysis. To avoid missing potential independent risk factors, the factors whose *P* value was less than 0.10 were considered to be statistically significant and were included in the multivariable analysis. Univariate logistic analysis showed that advanced age, premenopause, multiple parity and high expression of PRKCI and CSKN1A1 were associated with CIN progression. Using the above factors in the multivariable analysis, premenopause and high expression of PRKCI and CSKN1A1 were independent risk factors for CIN progression (Table 3).

**Table 2:** The clinical features and genes expressed in the CIN and normal groups

	normal	CIN	Z/X^2^	P
**age**	44.08 ± 10.00	41.71 ± 9.31	−1.480	0.139
gravidity	3.00 ± 1.76	3.46 ± 1.84	−1.536	0.124
**parity**	1.66 ± 0.85	1.67 ± 1.34	−0.561	0.561
HPV infection			26.552	**0.000**
positive	15	47		
negative	23	5		
**menopause**				
yes	9	9	0.558	0.455
no	29	43		
TCT				
CINII-(Normal/ASCUS)	28	10	/	**0.000**
CINII+(LSIL/HSIL/SCC)	16	36		
CTNNB1	0.85 ± 0.15	0.88 ± 0.24	−1.626	0.104
**TGFBR2**	1613.35 ± 369.73	665.56 ± 357.49	−2.916	**0.004**
**FOXO1**	0.04 ± 0.01	0.50 ± 0.30	−2.728	**0.006**
PRKCI	0.65 ± 0.22	0.56 ± 0.11	−1.675	0.094
CSKN1A1	211.45 ± 120.00	135.69 ± 81.39	−0.270	0.787
TP73	150.80 ± 44.11	75.39 ± 21.31	−1.805	0.071
MUC2	0.92 ± 0.25	1.89 ± 1.26	−0.343	0.732
PIK3CA	4.91 ± 0.87	7.28 ± 1.98	−1.005	0.315
CCND2	0.47 ± 0.19	1.40 ± 0.72	−0.556	0.579
**CTPB2**	40.18 ± 11.28	16.59 ± 6.14	−2.091	**0.037**

Similarly, the factors related to the occurrence of CIN identified in part 3.2, including HPV infection, CINII+ diagnosis by TCT, and the significantly differentially expressed genes TGFBR2, FOXO1, and CTBP2, were included in the univariate logistic analysis. Univariate analysis found that HPV infection, CINII+ diagnosis by TCT, high expression of FOXO1 and low expression of CTBP2 were associated with CIN. Including the above factors in the multivariable analysis revealed that HPV infection, CINII+ diagnosis by TCT, and low expression of CTBP2 were independent risk factors associated with CIN (Table 4).

**Table 3:** Logistic regression analysis of risk factors for CIN progression

	Univariate logistic analysis			Multivariate logistic analysis		
	HR	95% CI	P	HR	95% CI	P
**age**	1.089	1.034–1.146	**0.001**	0.986	0.886–1.097	0.794
**Premenopause**	6.248	2.256–17.303	**0.000**	11.36	1.175–117.976	**0.036**
parity	1.416	0.990–2.026	**0.057**	1.064	0.599–1.888	0.833
TGFBR2	1.000	1.000–1.000	0.174	/	/	**/**
FOXO1	0.404	0.079–2.053	0.274	/	/	**/**
**PRCKI**	2.113	1.212–3.683	**0.008**	3.363	1.153–9.810	**0.026**
**CSKN1A1**	1.001	1.000–1.001	**0.025**	1.001	1.000–1.002	**0.040**
CTBP2	1.006	0.999–1.012	**0.091**	1.005	0.988–1.023	0.534

### Establishment and evaluation of predictive random forest models

Based on the above results, different combinations of significant clinical factors and genes were selected to establish random forest models. Because model 13 only included one factor, it was not amenable to the random forest model method. Therefore, a total of 13 models were established for predicting the occurrence and progression of CIN (7 models for predicting CIN progression and 6 models for predicting CIN occurrence) (Table 5 and 6). To avoid missing potential independent risk factors, the factors whose *P* value was less than 0.10 were enrolled.

**Table 4:** Logistic regression analysis of risk factors for CIN occurrence

	Univariate logistic analysis			Multivariate logistic analysis		
	HR	95% CI	P	HR	95% CI	P
**HPV infection**	14.413	4.664–44.545	**0.000**	18.984	4.368–82.504	**0.000**
**CINII+**	6.300	2.481–15.995	**0.000**	9.785	2.525–37.921	**0.001**
TGFBR2	1.000	1.000–1.000	0.106	/	/	**/**
**FOXO1**	207.63	1.063–40,539.222	**0.047**	22.660	0.136–3789.024	0.233
CTBP2	0.992	0.983–1.001	**0.076**	0.987	0.977–0.997	**0.011**

**Table 5:** Random forest models for predicting CIN progression

NO.	Indicators	Factors	accuracy	AUC	OOB
1	All clinical features	age + menopause+HPV + gravidity+parity+TCT	65.85	67.75	36.59
2	Significant genes	TGFBR2 + CSKN1A1 + PRKCI+FOXO1 + CTBP2	73.17	86.75	29.27
**3**	Significant genes + significant clinical features	TGFBR2 + CSKN1A1 + PRKCI+FOXO1 + CTBP2+ menopause+parity+age	**75.61**	**86.25**	**29.27**
4	Genes as the risk factors in unvariable logistic analysis	CSKN1A1 + PRKCI+CTBP2	68.29	72.75	24.39
5	Genes as the risk factors in unvariable logistic analysis + Significant genes	CSKN1A1 + PRKCI+CTBP2+ menopause+parity+age	68.29	78.75	26.83
6	Genes as the independent factors in multivariable logistic analysis	CSKN1A1 + PRKCI	70.73	68.25	21.95
7	Genes as the independent factors in multivariable logistic analysis	CSKN1A1 + PRKCI+ menopause+parity+age	68.29	76.75	26.83

**Table 6:** Random forest models for predicting CIN occurrence

NO.	Indicators	Factors	accuracy	AUC	OOB
8	All clinical features	age + menopause+HPV + gravidity+parity+TCT	60.00	75.20	28.89
9	Significant genes	TGFBR2+ FOXO1 + CTBP2	71.11	70.54	26.67
10	Significant genes + significant clinical features	TGFBR2 + CTBP2 + FOXO1 + HPV + TCT	77.78	92.09	26.67
11	Genes as the risk factors in unvariable logistic analysis	CTBP2 + FOXO1	73.33	74.51	40.00
**12**	Genes as the risk factors in unvariable logistic analysis + Significant genes	**CTBP2 + FOXO1 + HPV + TCT**	**84.44**	**90.51**	**22.22**
13	Genes as the independent factors in multivariable logistic analysis	CTBP2	/	/	/
14	Genes as the independent factors in multivariable logistic analysis	CTBP2 + HPV + TCT	75.56	85.38	26.67

**Fig. 2:**
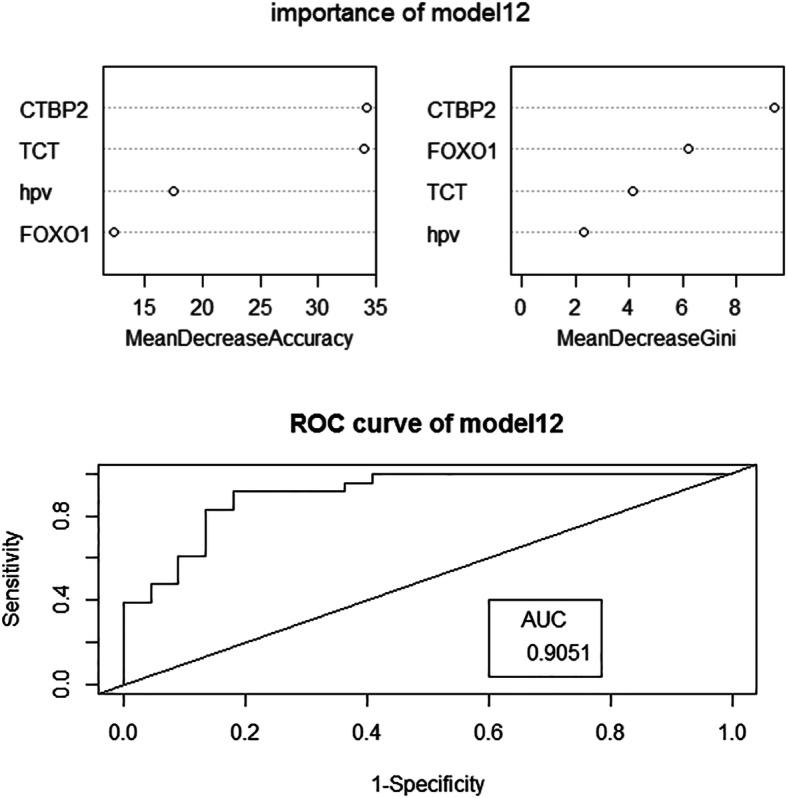
the weight of factors in model12. the ROC curve of model12

Models were assessed according to the accuracy rate, AUC and OOB error value of each. Among the 7 models predicting CIN progression, model 3 had the highest accuracy rate and the largest AUC, while the OOB error value was relatively small; therefore, model 3 was selected as the model for CIN progression (Fig. 1). Model 12 was adopted as the model for CIN occurrence (Fig. 2).

## Discussion

Currently, there are a large number of studies from across the world studying genes and biomarkers related to CIN progression and occurrence. However, the majority of studies report on single genes or single biomarkers that are associated with CIN, and only a few studies have combined multiple factors to predict CIN progression and occurrence. Mei Sze Tan et al. [5] screened 9 differentially expressed genes in cervical cancer and normal tissues using bioinformatics tools. However, this study only screened the genes in the data set and provided no experimental verification, and there was no demonstration of the expression of these genes in tissue specimens or analysis of the diagnostic value of CIN. Petra Biewenga et al. [6] used clinical cervical cancer tissue specimens and normal cervical tissue specimens to conduct experimental research and screened 9313 significant genes, but no further detailed analysis of these expressed genes was performed. However, it has been suggested that there are a great deal of significant genes in normal cervical tissues, CIN tissues and SCC, which lays the foundation for multifactor combined diagnosis.

### The genes and pathways related to the occurrence and progression of CIN

**Fig. 3:**
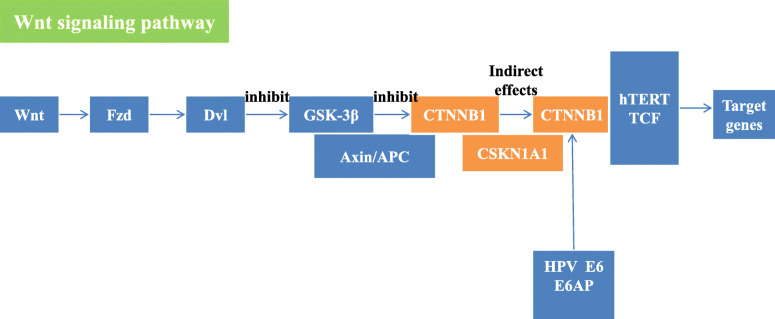
the roles of CSNK1A1 in Wnt signaling pathway

**Fig. 4:**
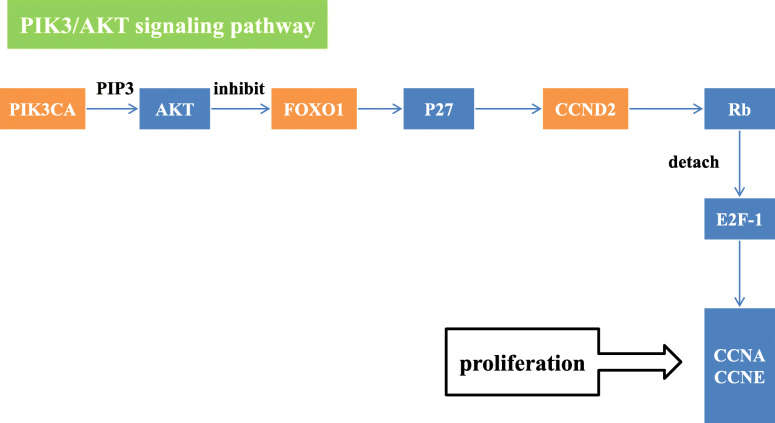
the roles and locations of PIK3CA and FOXO1 in HPV infection pathway

The HPV infection pathway summarizes the mechanism of HPV infection and the carcinogenic process. The HPV infection pathway includes 11 subpathways: the Wnt signaling pathway, mTOR signaling pathway, apoptosis pathway, NFKB signaling pathway, P53 signaling pathway, JAK/STAT signaling pathway, Notch signaling pathway, PI3K/Akt signaling pathway, Toll-like receptor signaling pathway, focal adhesion pathway and antigen processing and presentation pathway. In this study, FOXO1, CSKN1A1 and CTBP2 were significantly differentially expressed genes located in the HPV infection pathway. Among them, the significant genes CSKN1A1 and CTBP2 were located in the Wnt signaling pathway. HPV E6 can activate the Wnt signaling pathway, thereby causing immortalization of cervical epithelial cells [7]. In addition, HPV E6 acts on the gene Dvl, which is located upstream of the Wnt signaling pathway. The Dvl gene is overexpressed in cervical squamous carcinoma cells and plays a key role in the carcinogenesis of cervical epithelial cells [8]. While experimental results indicated that CSKN1A1 is located downstream of Dvl, it was speculated that in the progression of CIN, CSKN1A1 was affected by HPV E6 so that the cells acquired immortality(Fig. 3). CTBP2 has not been reported to be related to cervical diseases, and its role in the HPV infection pathway is unknown. In studies of gynecological tumors, L Barroilhet et al. [9]. pointed out that CTBP2 is overexpressed in ovarian cancer cells and that CTBP2 can downregulate the target gene of the Wnt signaling pathway and promote the carcinogenesis of ovarian epithelium, but its role in cervical cancer needs further study. FOXO1 is located in the PI3K/Akt signaling pathway. The PI3K/Akt signaling pathway can be activated by HPV E7, which can inactivate Rb and promote the occurrence of HSIL [10]. HPV E7 can upregulate the expression of FOXO1, which serves as the upstream gene of Akt, but Akt can inhibit the expression of FOXO1, so HPVE7 can indirectly inhibit the expression of FOXO1. In this study, FOXO1 expression was significantly lower in cervical cancer tissues than it was in normal tissues, and the FOXO1 gene was located upstream of the Rb gene in the PI3K/Akt signaling pathway. Therefore, low expression of the FOXO1 gene may be related to Rb inactivation(Fig. 4).

**Fig. 5:**
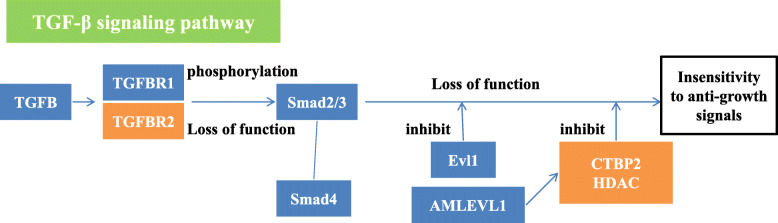
the roles of TGFBR2 and CTBP2 in TGFB signaling pathway

**Fig. 6:**
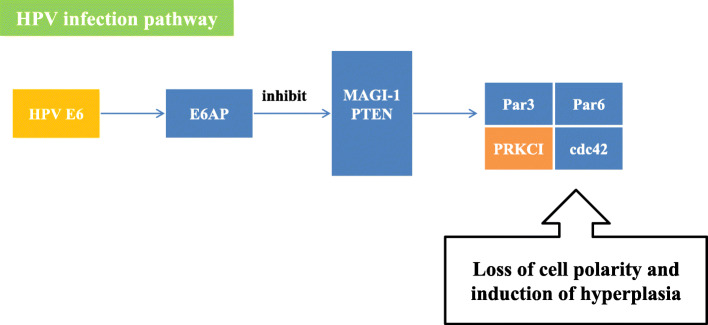
the role and location of PRKCI in HPV infection pathway

The main function of the Hippo signaling pathway is to control the normal size of organs. In the process of cervical carcinogenesis, the expression of the core gene of this pathway, YAP, is upregulated with the progression of cervical lesions [11]. Excessive activation of YAP increases the susceptibility of cervical epithelial cells to HPV, and YAP and HPV work together to promote carcinogenesis of cervical epithelium cells [12]. In this study, PRKCI and TGFBR2, which are located in the Hippo signaling pathway, were significantly differentially expressed genes. TGFBR2 is located upstream of YAP and inhibits the formation of apoptotic precursor proteins(Fig. 5). In the SCC group, the expression of TGFBR2 was significantly higher than it was in the CIN group. According to the experimental results, it is speculated that the overexpression of TGFBR2 inhibited the apoptosis of cervical epithelial cells, and together with the synergistic effect of HPV, carcinogenesis of cervical epithelium cells was promoted. Compared with normal cervical tissue, the expression of TGFB2 in CIN tissue is significantly lower, and it decreases with the progression of CIN [13]. TGFBR2 is a receptor protein of TGFB2, and the decreased expression of TGFB2 is likely to cause a similar change in TGFBR2. Previous studies have revealed that cervical cancer cases with low expression of TGFBR2 have a poor prognosis and have confirmed that TGFBR2 can inhibit the cell cycle process at the G1/S stage through the TGFB/Smad pathway, while low expression of TGFBR2 can alleviate the inhibitory effect of this pathway, thereby speeding up cervical cancer cell progression from the G1 phase to the S phase and resulting in cell proliferation [14]. TGFBR2 works via different pathways in the process of initiation and progression of CIN. Kyung-Hee Kim et al. [15] reported that overexpression of the YAP gene in lung adenocarcinoma can result in the phosphorylation of PRKCI, which upregulates the expression of PRKCI, suggesting a high pathological grade and an unfavorable prognosis. PRKCI likely inhibits the recruitment of immune cells in the microenvironment of ovarian cancer by regulating the activity of YAP1 through the Hippo signaling pathway, resulting in immunosuppression and promoting tumor growth [16]. There are few reports of PRKCI and its role in the carcinogenic mechanisms of cervical cancer(Fig. 6). Femi OF et al. [17] demonstrated that a PRKCI mutation is related to the occurrence of cervical cancer, but the specific mechanism remains unclear(Fig. 6).

### Clinical factors related to the occurrence and progression of CIN

In this study, the proportion of premenopausal cases of CIN was significantly higher than that of SCC cases, and logistic analysis found that premenopause was one of the independent risk factors for the progression of CIN. Chen et al. [18] studied patients with CIN who relapsed after receiving cervical conization or LEEP treatment, and the reoccurrence rate of premenopausal patients was significantly higher than that of menopausal patients, which is consistent with this study. However, Renata B et al. [19] reported that postmenopausal CIN patients were more prone to interstitial infiltration and progression to invasive cervical cancer. Therefore, it is still unclear whether menopause has any effect on the progression of CIN. According to the results of this study, it could be speculated that patients without menopause were younger, had more active sexual activity and were more likely to have persistent HPV infection [20]. At the same time, the level of endogenous estrogen in premenopausal females is higher [21], and the high level of estrogen promotes the transcription and integration of HPV and the degradation of the host cell P53 protein, thereby causing cervical epithelial cells to become cancerous [22]. Moreover, young premenopausal women are more likely to take oral hormonal contraceptives, and oral hormonal contraceptives are also one of the risk factors for the progression of CIN [23].

Compared to CIN patients, the average age of SCC patients was greater, and the parity was significantly more than that of the CIN cases. For women younger than 25 years old, regardless of the level of cervical lesions, the rate of spontaneous regression was 1.4 times higher than that of women older than 50 years old [24]. Christine Bekos [25] obtained similar results; the proportion of women over 40 years old who experienced CIN progression was significantly higher than the proportion who were younger than 40 years, and for every extra 5 years of age, despite cervical lesion grades, the rate of spontaneous regression decreased by 21%. The results of this study showed that the average age for patients with SCC is significantly greater than that of CIN patients, suggesting that age is likely to be related to the progression of CIN. As age increases, immune function declines, leading to persistent HPV infection. In addition, the parity of patients with SCC was significantly greater than that of patients with CIN. Among women with persistent HPV infection, the greater the number of deliveries there were, the greater the risk of developing high-grade cervical lesions was [26]. High parity is a risk factor for cervical cancer [27]. Especially for women who are elderly and have high parity, HSIL is more likely to progress [28]. The results of this study are consistent with those reported in previous research.

Compared to patients in the normal cervical group, the proportions of HPV positivity and CINII+ TCT results in CIN cases were significantly higher than they were in the normal cervical group. In model 12, the TCT results had a large impact on the results. This shows that TCT examination played an important role in the diagnosis of CIN. HPV and TCT play an important role in diagnosing CIN and identifying CIN and SCC. Although the results of TCT will cause false negatives due to the different methods of the operators, the accuracy of TCT in the diagnosis of cervical diseases has been significantly improved compared to traditional cervical smears [29]. Among HPV-negative women, the proportion of women with normal TCT results and cervical biopsies who experienced CINII + after 15 years of follow-up was only 4.8%. However, 46.2% of women with TCT results of HSIL+ experienced disease progression [30]. Moreover, HPV is an important factor in the occurrence of CIN and cervical cancer [2], and TCT combined with HPV detection has greatly promoted the early diagnosis of cervical disease. Hence, patients with HPV infection and TCT results with CINII+ should undergo further examination and follow-up to prevent the occurrence and progression of cervical lesions.

### The predictive random forest models

The random forest model consists of multiple decision trees, and there is no correlation between decision trees. When a new input sample enters, it will be judged by each decision tree. The random forest model is capable of preventing fitting, has low requirements of the data set, and has strong adaptability, making it suitable for nonlinear data. In this study, a random forest algorithm was used to build random forest models. Then, we choose the best models according to the accuracy, AUC value and OOB error value.

**Fig. 7:**
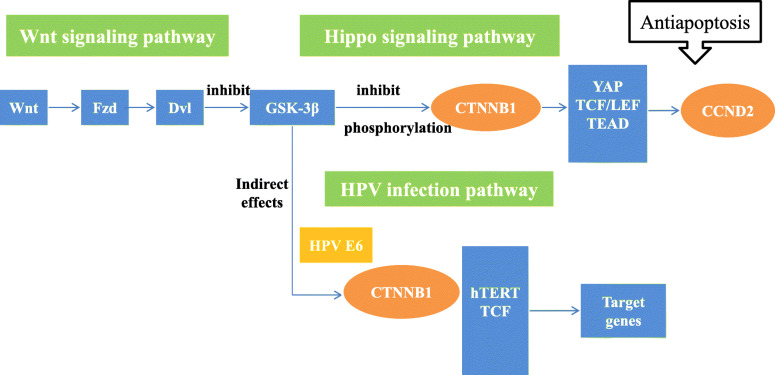
the roles and locations of CCND2 and CTNNB1 in Hippo signaling pathway and HPV infection pathway

Regarding the random forest models of CIN progression, model 3 had the highest accuracy and AUC value, and the OOB error value was relatively small. Therefore, model 3 was chosen as the predictive model for CIN progression. In model 3, CSNK1A1 and PRKCI had a great impact on the result. Moreover, these two genes were also significantly differentially expressed genes during the progression of CIN. In the HPV infection signaling pathway, CSNK1A1 can cause cell polarity loss through the action of HPVE6. However, there is no research on the expression of CSNK1A1 and cervical diseases. Most of the research on CSNK1A1 focuses on hematological malignancies. Overexpression of CSNK1A1 can promote the proliferation and survival of tumor cells by downregulating the expression of CTNNB1 in myeloma [31]; CSNK1A1 and CTNNB1 both function in the classic Wnt/β-catenin signaling pathway. CSNK1A1 inhibits the canonical Wnt/β-catenin signaling pathway by promoting the degradation of CTNNB1, thereby promoting tumor cell growth [32]. However, in this study, the expression of CSNK1A1 in cervical cancer tissue was significantly higher than it was in CIN tissue, but the expression of CTNNB1 in CIN and SCC tissues was not significantly different. According to the experimental results, it is speculated that the overexpression of CSNK1A1 has no effect on CTNNB1 during the progression of CIN, so it may not promote cell proliferation or even malignancy through pathways other than the Wnt/β-catenin signaling pathway(Fig. 7). PRKCI is in the Hippo signaling pathway, but the mechanism by which it leads to CIN and cervical cancer is unknown. According to previous studies, overexpression of YAP in this pathway may lead to upregulation of PRKCI, which eventually results in carcinogenesis. PRKCI has been confirmed to be overexpressed in many solid tumors. In the study of gynecological tumors, the expression of PRKCI in ovarian cancer tissues was significantly higher than it was in normal tissues, and it enhances the invasion and proliferation ability of ovarian cancer cells [33]. The experimental results of this study showed that the expression of PRKCI in cervical cancer tissue was significantly higher than it was in CIN tissue, which may be related to the progression of CIN. However, more research is needed to uncover the mechanism.

For the random forest model of CIN occurrence, the accuracy rate and AUC value of model 12 are the largest, and the OOB error value is the smallest. Therefore, model 12 was chosen as a predictive model for the occurrence of CIN. CTBP2 has the largest impact on the model 12. CTBP2 is in the classic Wnt/β-catenin signaling pathway, which is one of the subpathways of the HPV infection signaling pathway; however, the role of CTBP2 in the HPV infection signaling pathway is unknown. In this study, the expression of CTBP2 in normal cervical tissue was higher than it was in CIN tissue. Surprisingly, compared with normal cervical tissue, SCC tissue expresses CTBP2 at much higher levels. Overexpression of CTBP2 might be linked to the progression of CIN. CTBP2 has not yet been reported in cervical diseases, but some studies have found that CTBP2 is associated with a variety of solid tumors. CTBP2 is overexpressed in non-small cell lung cancer and promotes tumor cell invasion and proliferation through the classic Wnt/β-catenin signaling pathway [34]. Additionally, CTBP2 is related to angiogenesis in prostate cancer cells, and silencing CTBP2 can promote prostate cancer cell apoptosis [35]. Similar results were obtained in this study, in which CTBP2 was overexpressed in SCC tissues. Overexpression of CTBP2 can also inhibit the expression of genes located downstream of the classic Wnt/β-catenin signaling pathway, such as CTNNB1 [34]; however, in this experiment, CTNNB1 was not inhibited by overexpressed CTBP2, indicating that the overexpression of CTBP2 might not promote the malignant transformation of cervical cells through Wnt/β-catenin signaling pathway, and it might use other pathways instead, leading to the occurrence and progression of CIN. Another study suggested that CTBP2 can promote epithelial-mesenchymal transition (EMT) [36], and EMT is the key process of epithelial cell carcinogenesis. In addition, CTBP2 can promote the replication and proliferation of adenovirus in 293 T cells [37]. Since most CIN and SCC cases involve persistent HPV infection, it can be inferred that overexpression of CTBP2 may be related to HPV infection and may promote the malignant transformation of cells by promoting the replication and proliferation of HPV in cells.

TGFBR2 and FOXO1 were two other significant genes that might be related to the occurrence and progression of CIN. Among the tested models, model 3 incorporates TGFBR2 and FOXO1, and model 12 incorporates FOXO1. These two genes influence the progression and occurrence of CIN. There are few reports on the correlation between TGFBR2 and cervical diseases. Cai et al. [38] pointed out that miR-17-5p promotes the development and metastasis of cervical cancer by upregulating TGFBR2. In contrast, Yang et al. [14] reported that the downregulation of TGFBR2 suggests a poor prognosis for cervical cancer. In the early stages of carcinogenesis, TGF-β plays an inhibiting role, while in the later stages of carcinogenesis, tumor cells lose sensitivity to TGF-β signaling and take advantage of TGF-β signaling to promote cellular EMT [39]. This study found that the expression of TGFBR2 in CIN tissue was significantly lower than it was in normal cervical tissue and SCC tissue. It is speculated that when precancerous lesions of cervical epithelial cells occur, the expression of TGFBR2 is downregulated, and TGF-β signaling does not function as an inhibitor of cancerous cells. When CIN progresses to SCC, cells and tissues become resistant to TGF-β signaling, and TGFBR2 is upregulated and promotes the progression of CIN. In addition, several studies have described that TGFBR2 is closely related to EMT during carcinogenesis. In immortalized cervical epithelial cells, overexpression of TGFRB2 promotes EMT and malignant transformation of immortalized cervical epithelial cells [40]. TGFBR2 may also accelerate the malignant transformation of cervical epithelial cells and the progression and initiation of CIN by promoting EMT.

FOXO1 plays an important role in the occurrence and progression of CIN. FOXO1 is considered to be a tumor suppressor gene. Overexpression of FOXO1 in vitro can inhibit the growth and proliferation of cervical cancer cells. Moreover, the prognosis of cervical cancer with FOXO1 overexpression is more satisfactory [41]. In contrast, the downregulation of FOXO1 promotes the invasion and metastasis of cervical cancer cells [42]. The role of FOXO1 in the development of cervical cancer is still controversial. The results of this study showed that the expression of FOXO1 in SCC tissue was significantly lower than it was in CIN tissue, but the expression of FOXO1 in CIN tissue was significantly higher than it was in normal cervical tissue. According to the experimental results and pathway information, during the progression of CIN to SCC, FOXO1 functions in the PI3K-Akt signaling pathway, which is a part of the HPV infection signaling pathway. This pathway is related to cell proliferation, and upstream genes can inhibit the expression of FOXO1 to promote cell proliferation. Thus, the low expression of FOXO1 may be linked to the excessive proliferation of cells, which may promote the progression of CIN to cervical cancer. However, some studies reported that inhibiting FOXO1 expression could inhibit cervical cancer growth [43]. Chay et al. [44] reported that the expression of FOXO1 in cervical cancer tissue and CIN tissue is significantly higher than it is in normal cervical tissue and that FOXO1 overexpression is an independent risk factor for poor prognosis of cervical cancer. Concerning the mechanism of CIN occurrence, FOXO1 might have a tendency to be overexpressed to inhibit the progression of lesions to cervical cancer. The overexpression of FOXO1 in CIN might be a warning of the progression of CIN.

This study has some limitations. First, this is a single-center retrospective analysis with a small sample size. Second, there are some errors in the experimental results. In the future, it will be necessary to expand the sample size and improve the experimental methods to fully assess risk factors related to the occurrence and progression of CIN.

## Conclusions

In summary, the high expression of PRKCI and CSKN1A1 and premenopause are independent risk factors for the progression of CIN to SCC. Meanwhile, a random forest method combining clinical features and significantly differentially expressed genes predicted the occurrence and progression:n of CIN. For patients who are premenopausal, are older, have CINII+ results from TCT, have high parity, have high expression of TGFBR2, CSKN1A1, PRKCI, and CTBP2, and exhibit low expression of FOXO1, more attention should be paid to CIN progression. For women with HPV infection, CINII+ results from TCT, high FOXO1 expression and low CTBP2 expression, it is necessary to be vigilant regarding the occurrence of CIN.

## Supplementary information

**Additional file 1: Table S1.** Sequences of primers.

**Additional file 2.**

**Additional file 3.**

## Data Availability

The datasets used and/or analyzed during the current study are available from the corresponding author on reasonable request.

## Abbreviations

CIN: Cervical intraepithelial neoplasia
KEGG: Kyoto Encyclopedia of Genes and Genomes
RT-qPCR: Real-time fluorescence quantitative polymerase chain reaction
SCC: Cervical squamous cell cancer
HPV: Human papillomavirus
OOB error: Out-of-bag error
ROC: Receiver operating characteristic curve
AUC: Area under the curve of ROC
