# Supplementary material for: Establishment of multifactor predictive models for the occurrence and progression of cervical intraepithelial neoplasia
Source: BMC Cancer. 2020 Sep 29;20:926. doi: 10.1186/s12885-020-07265-7 (PMC7523359; doi:10.1186/s12885-020-07265-7)
Supplement: Supplementary file 1 — Additional file 1: Table S1. Sequences of primers. [file 12885_2020_7265_MOESM1_ESM.pdf]

Table S1 Sequences of primers

Gene name	Forward sequences	Reverse sequences
CCND2	ACCTTCCGCAGTGCTCCTA	CCCAGCCAAGAAACGGTCC
CTNNB1	CATCTACACAGTTTGATGCTGCT	GCAGTTTTGTCAGTTCAGGGA
PRKCI	GACAACGAACAGCTCTTCACC	CCAGGACGTTCTGGTACACA
PIK3CA	AGTAGGCAACCGTGAAGAAAAG	GAGGTGAATTGAGGTCCCTAAGA
FOXO1	AAGATGACCGCTCTGACATCA	CTTATAGACCTCAGCAAAGCGAC
MUC2	AAGATGACCGCTCTGACATCA	CTTATAGACCTCAGCAAAGCGAC
TGFBR2	AAGATGACCGCTCTGACATCA	CTTATAGACCTCAGCAAAGCGAC
TP73	CGGGCCATGCCTGTTTACA	TGTCCTTCGTTGAAGTCCCTC
CSKN1A1	AGTGGCAGTGAAGCTAGAATCT	CGCCCAATACCCATTAGGAAGTT
CTBP2	ATCCACGAGAAGGTTCTAAACGA	CCGCACGATCACTCTCAGG
β-actin	CTCCATCCTGGCCTCGCTGT	GCTGTCACCTTCACCGTTCC
