# Supplementary material for: Establishment of multifactor predictive models for the occurrence and progression of cervical intraepithelial neoplasia
Source: BMC Cancer. 2020 Sep 29;20:926. doi: 10.1186/s12885-020-07265-7 (PMC7523359; doi:10.1186/s12885-020-07265-7)
Supplement: Supplementary file 3 — Additional file 3. [file 12885_2020_7265_MOESM3_ESM.docx]

| Sequences of primers | | |
| --- | --- | --- |
| Gene name | Forward sequences | Reverse sequences |
| CCND2 | ACCTTCCGCAGTGCTCCTA | CCCAGCCAAGAAACGGTCC |
| CTNNB1 | CATCTACACAGTTTGATGCTGCT | GCAGTTTTGTCAGTTCAGGGA |
| PRKCI | GACAACGAACAGCTCTTCACC | CCAGGACGTTCTGGTACACA |
| PIK3CA | AGTAGGCAACCGTGAAGAAAAG | GAGGTGAATTGAGGTCCCTAAGA |
| FOXO1 | AAGATGACCGCTCTGACATCA | CTTATAGACCTCAGCAAAGCGAC |
| MUC2 | AAGATGACCGCTCTGACATCA | CTTATAGACCTCAGCAAAGCGAC |
| TGFBR2 | AAGATGACCGCTCTGACATCA | CTTATAGACCTCAGCAAAGCGAC |
| TP73 | CGGGCCATGCCTGTTTACA | TGTCCTTCGTTGAAGTCCCTC |
| CSKN1A1 | AGTGGCAGTGAAGCTAGAATCT | CGCCCAATACCCATTAGGAAGTT |
| CTBP2 | ATCCACGAGAAGGTTCTAAACGA | CCGCACGATCACTCTCAGG |
| β-actin | CTCCATCCTGGCCTCGCTGT | GCTGTCACCTTCACCGTTCC |
